# Surprisingly high prevalence rates of severe psychological distress among consumers who purchase loot boxes in video games

**DOI:** 10.1038/s41598-022-20549-1

**Published:** 2022-09-27

**Authors:** Aaron Drummond, Lauren C. Hall, James D. Sauer

**Affiliations:** 1 School of Psychology, Massey University, Palmerston North, 4424 Manawatu New Zealand; 2 School of Psychological Sciences, University of Tasmania, Hobart, Australia

**Keywords:** Psychology, Human behaviour

## Abstract

Loot boxes are randomised rewards available in some video games, often purchasable for real-world money. Loot boxes have been likened to conventional forms of gambling and may satisfy legal requirements to be considered bona fide gambling in some jurisdictions. Research has consistently shown that people with problem gambling symptoms report spending more on these mechanisms than people without such symptoms. However, a significant gap in our current understanding is whether engaging with these mechanisms is associated with harm. Here we examine the prevalence rates of severe psychological distress among purchasers of loot boxes relative to non-purchasers. A reanalysis of two cross-sectional surveys collected online via online collection platforms. Participants were 2432 Aotearoa New Zealand, Australian, and United States residents recruited through online survey. Our results show that purchasers of loot boxes are at approximately 1.87 times higher risk of severe psychological distress on a standardised clinical screening tool than people who do not purchase loot boxes. These relative risk rates are not due to gender, age, spending on other video game related purchases, or problem gambling symptoms. Individuals who purchased loot boxes appeared to also have higher risk of severe psychological distress irrespective of demographic characteristics or problem gambling status. Loot boxes appear to be associated with significantly higher risk of experiencing psychological harm even for players without problem gambling symptoms.

## Introduction

Loot boxes are randomised rewards in some video games, often available for purchase with real world money^1^. Almost 40% of popular PC (Steam) games and 60% of popular smart phone games contain loot boxes^2^. Loot boxes generated approximately $15 billion US in 2020 alone^3^. The randomised nature of loot box rewards, and their delivery on an intermittent reward schedule, has prompted concerns that they are psychologically akin to conventional gambling^1,4,5^.

The structural characteristics of loot boxes appear to be psychologically similar to conventional gambling by involving an optional monetary purchase of an unknown, chance-based outcome, resulting in some players winning and others losing^1,4^. It is perhaps these similarities to conventional gambling which lead participants with problem gambling symptoms to typically report spending more on loot boxes than participants without such symptoms^6–10^; a robust small-moderate relationship confirmed by meta-analysis^11^, and one that is stronger for adolescents than adults^12,13^. Further, when loot boxes are removed from a game, it is primarily problem gamblers who report reduced spending^14^. Interestingly, the relationship between problem gambling symptoms and purchasing does not occur for other, less immediately available randomised purchases (e.g., collectable card games^15^). This differential association with psychopathology implies that some randomised purchases—such as loot boxes—have a greater potential for harm than others (e.g., collectable card games).

Moreover, virtual items have value to players, with some players spending substantial amounts of real-world money to acquire them^16^. These virtual items are sometimes traded in legitimate marketplaces^16^ and, when legitimate marketplaces are unavailable, grey marketplaces have emerged^16^. Recently, black market economies have also developed, with some players spending in excess of 1000 euros per item to bypass the randomised loot box systems and purchase the rarest items^17^. As virtual items obtained from loot boxes have value to players, they may satisfy the requirements of a prize, making them potentially suitable for regulation under some jurisdictions’ existing gambling legislation^16^. Some policymakers have opted to regulate these systems under existing gambling legislation, while others have implemented warning labels or odds disclosure requirements^18^.

### Loot boxes and harm

A significant gap in the literature, however, is whether loot boxes are associated with harm^19^. Although disproportionate purchasing of loot boxes by a vulnerable group of problem gamblers might imply the existence of harm, this does not conclusively show that loot boxes are associated with harm per se^19^. Here, we combine two large independent datasets^7,8^, and make important strides toward addressing this issue by demonstrating that those who have purchased loot box have surprisingly high rates of severe psychological distress—as indexed by a standardised psychiatric screening measure, reliably predictive of clinical anxiety and depression disorders. Further, these rates are not due to gender or age. These rates exceed the rates observed for people who spend money on other kinds of video game related purchases including games, downloadable content, and non-randomised in-game virtual items. Thus, the higher rates of severe psychological distress are unlikely to be attributable to the population of gamers generally, or indicate that loot box purchasing represents a generalised form of “retail therapy” (because the higher rates of distress are not associated with other video game purchases). Most importantly, these rates are not explained by problem gambling symptoms: even those without problem gambling symptoms may experience psychological harms from engaging with loot boxes.

### Psychological distress

In two separate studies we included the Kessler-10 Psychological Distress Scale to assess whether loot box spending was associated with psychological distress^7,8^. The Kessler-10 asks participants to indicate how frequently during the past 30 days they have experienced certain symptoms of psychological distress—for instance “About how often did you feel so nervous that nothing could calm you down?”. Weak correlations were observed between loot box spending and psychological distress in both of these studies, a finding we initially interpreted as being unlikely to be clinically meaningful due to the small effect size. To foreshadow, the reanalyses presented here imply we were wrong.

We had two primary motivations for reanalysing the psychological distress data of our previous two studies. First, as noted earlier, the previous analyses of psychological distress were designed to determine whether the continuous measures of psychological distress were correlated with loot box spending. Although interesting, this does not speak to the issue of clinical harm raised by critics of loot box regulation per se^19^. To understand whether loot box purchasing was associated with clinical harm in our sample, a more important question was whether participants were exceeding thresholds indicating clinically meaningful levels of distress on the Kessler-10. Indeed, the primary purpose of the Kessler-10 is to identify respondents that exceed normed population scores for psychological distress, and who are likely to be at risk for a diagnosable clinical psychological disorder. The Kessler-10 is widely employed as a validated standardized screening measure to identify individuals likely to have serious mental illness/psychopathology, such as a clinical affective disorder (e.g., anxiety/depression)^20,21^. Although concerns exist about the use of the Kessler-10 in culturally diverse settings^22^, it has been demonstrated as an effective screening tool for affective disorders in US^20,21^, Australian^23^, and Aotearoa New Zealand^24^ populations (the three populations sampled in the datasets re-analysed herein). The Kessler-10 has excellent discrimination for identifying affective disorders^20^. Taking a Bayesian probability updating perspective, assuming that any individual in the population has a base-rate for a mental disorder of around 20%, a very high score (30 or more) on the Kessler-10 increases the posterior probability of an individual having a diagnosable affective disorder to over 80%^23^. For this reason, extreme scores on the Kessler-10 are a valid proxy for population prevalence estimates for serious mental illness^24^. Second, recent evidence suggests that Kessler Psychological Distress Scales may have poor sensitivity to change when employed as a continuous outcome measure, and therefore may have limited utility in detecting subtle differences in psychological distress^25^. For both of these reasons, and given the need to better understand the potential for harm associated with loot box purchasing^19^, we evaluated whether our data showed any evidence for serious mental distress for individuals who had purchased loot boxes based on a categorical assessment of whether reported distress level met thresholds to be considered clinically meaningful.

## Methods

### Participants

We combined data from two different datasets collected to examine the relationship between loot box purchasing and problem gambling symptoms^7,8^. The first dataset was collected in 2019, and contained 436 United States, 433 Australian and 419 Aotearoa New Zealand participants using Qualtrics’ survey targeting tool. The second dataset was collected in 2020 using Prolific Academic, and contained 930 United States, 173 Australian and 41 Aotearoa New Zealand participants. There were no significant differences in psychological distress across these two datasets, χ^2^ (1, 2464) = 0.05, p = 0.826. When combined, these datasets provided us with a sample of 1366 United States respondents, 606 Australian respondents, and 460 Aotearoa New Zealand respondents. One thousand three hundred and fifteen (1315) participants identified as female, 1076 identified as male, and 41 identified as other. As in the original studies, we excluded participants who failed any attention check. For both datasets, ethics approval was obtained for human data collection from Massey University’s Human Ethics Southern B Committee, Approval number SOB 19/11. In both studies all research was performed in accordance with the relevant guidelines and regulations, and participants gave informed consent to participate in the study. We included dataset as a factor in all analyses to ensure that the different studies and the associated time-periods for data collection did not systematically alter our results. This was especially important as the second dataset was gathered during the COVID-19 pandemic.

In both studies, participants answered a series of questions about their gambling behaviours, loot box purchasing and spending behaviours, and their psychological distress over the past month. In both studies, the core association of interest was the association between problem gambling symptomatology and loot box spending. Participants then reported their gender (male, female, non-binary, other, prefer not to disclose) and age.

### Measures

#### Age

In both studies, participants indicated their age by responding to the question ‘What is your age?’ using a drop down menu to indicate their current age in years.

#### Gender

In both studies, participants were asked “What is your gender”. Response options were male, female, non-binary, prefer not to say, other (please specify).

#### Dataset

Dataset was dummy coded to ensure that differences between the two studies were not contributing systematic noise to the data. Dataset was coded as either Study 1, or Study 2.

#### Psychological distress

Psychological distress was measured using the validated Kessler-10 psychological distress scale^20,21^. Exceeding a score of 30 on this scale indicates severe psychological distress and high risk for an affective disorder^20,21^. Participants were coded as either having exceeded this criterion or not. Internal reliability of this scale was high (α = 0.915)^7^.

#### Problem gambling symptoms

Problem gambling symptoms were measured using the Problem Gambling Severity Inventory (PGSI)^26^. This inventory produces a continuous measure of problem gambling symptomology which can be used to classify gamblers into four categories, non-problem gamblers, low-risk gamblers, moderate risk gamblers, and problem gamblers. Participants who score 0 on the scale are considered non-problem gamblers; low risk gamblers score 1–2 on the PGSI; moderate risk gamblers = 3–7 on the PGSI and problem gamblers score 8 or higher on the PGSI^26^.

#### Loot box purchasing

Participants reported how much money they had spent on loot boxes in the past month in their own currency. All currencies were then converted to US dollars according to the currency conversion rates at the time. For Dataset 1, we converted all spending into US dollars (USD) on the 2nd of October, 2019, using the listed currency conversion rates of the day using Google’s currency conversion. For Australian purchases, this resulted in the spending data being 0.67 times the reported amount, and for Aotearoa New Zealand purchases, this resulted in the spending data being 0.62 times the reported amount^7^. For Dataset 2, we converted all currencies into US dollars on the 21st of April, 2020 using the listed currency conversion rates of the day using Google’s currency conversion, which were the following exchange rates: $USD = 0.63*AUD; $USD = 0.60*$NZD^8^.

#### Other forms of game-related purchasing

Participants were asked to report how much money they had spent on buying video games, on downloadable content, and on other forms of non-randomised in game content in the past month in their own currency. As reported above in the loot box spending questions, all currencies were then converted to US dollars according the currency conversion rates at the time of data collection using the conversion rates specified above.

#### Relative risk estimates

For all chi-square analyses incorporating no statistical control variables, relative risk was directly calculated from the percentages by JAMOVI version 1.6.1.5. For binomial logistic regression analyses, JAMOVI only provides Odds Ratios. Therefore, we converted these Odds Ratios to Relative Risks using the formula detailed in Grant^27^. Manual estimation of the Relative Risks using the estimated marginal means provided by JAMOVI yielded almost identical results.

## Results

First we examined whether the proportion of people scoring within the very high range on the Kessler-10 was higher for those that purchased loot boxes than those that did not. Many players that purchase loot boxes spend less than $15–25 US per month on loot boxes^7,8^. Thus, we might not expect differences in distress between individuals that purchased loot boxes and those that did not. If loot box purchasing is associated with distress, it seemed most likely that only the highest loot box spenders would be at elevated risk of severe distress. We were therefore surprised to find that the *Relative Risk* (*RR*) of being in the very high distress range increased markedly for those that purchased loot boxes (i.e., those who reported spending *any* money on loot boxes) compared to those that did not. For players that did not purchase loot boxes, 23.5% of players were suffering from severe psychological distress. In contrast, 43.9% of players that purchased loot boxes suffered from severe psychological distress. Thus, those that purchased loot boxes were at 1.87 [1.62, 2.16] times the risk of experiencing severe distress than non-purchasers, χ^2^ (1, 2462) = 61.0, *p* < 0.001. When included in a binomial logistic regression analysis with age as a covariate and gender and dataset as factors (see Table 1), the players that purchased loot boxes remained at significantly greater risk of severe psychological distress than players that did not purchase loot boxes, *RR* = 1.79 [1.53, 2.05], *p* < 0.001.The unexpectedly large size of these effects suggested to us the possibility that these differences may be attributable to a third variable. We subsequently examined (a) whether the effects were partially or fully attributable to a ‘retail therapy’ effect of purchasing patterns more generally, and (b) whether the previously observed association between loot box spending and problem gambling symptomology might partially or fully account for the observed associations.

**Table 1:** Binomial linear regression results with age as a covariate and gender and dataset as factors.

Predictor	β	Z	Relative risk [95% CI]	*p*
Age	− 0.45^1^	− 7.73	n/a^2^	< 0.001
Gender: female^3^	0.32	3.24	1.27 [1.10, 1.45]	0.001
Gender: non-binary^3^	1.02	2.33	1.96 [1.13, 2.85]	0.020
Gender: “prefer-not-to-say”^3^	0.10	0.15	1.08 [0.35, 2.41]	0.878
Gender: other^3^	− 0.01	− 0.01	0.99 [0.13, 3.19]	0.999
Dataset	− 0.20	− 1.94	0.86 [0.74, 1.00]	0.052
Loot box purchased	0.86	6.73	1.79 [1.53, 2.05]	< 0.001

^1^Age converted to Z-score to generate β.

^2^Relative Risk inappropriate for age as a continuous covariate.

^3^Comparison group: male.

**Figure 1:**
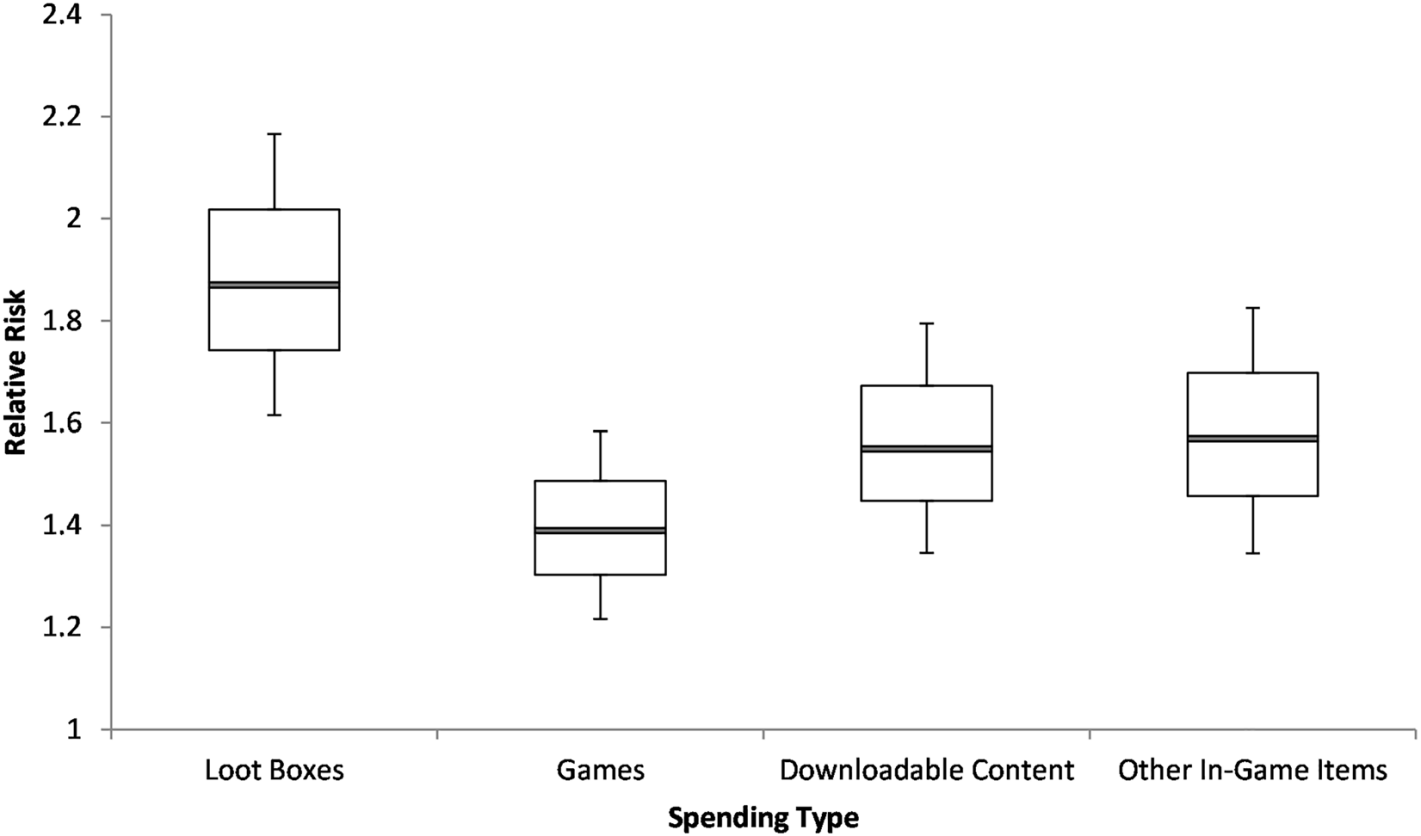
Relative risk of those that purchased loot boxes, video games, downloadable content, and other in-game items scoring 30 or more on the Kessler-10 psychological distress scale compared to those that did not purchase such content. White bars represent 68% confidence interval (1 SD). Error bars represent 95% confidence intervals.

First, we considered whether this effect might indicate a more general ‘retail therapy’ effect, where gamers experiencing severe distress might be spending more, via their hobby, in an attempt to improve their mood^28^. If so, gamers who spend money on *any* gaming-related purchases may be more likely to score in the very high range of the Kessler-10. To test this, we examined the relationship between Kessler-10 scores and spending on non-randomised video game purchases—namely games themselves, downloadable content (a form of non-randomised video game expansion), and other non-randomised in-game purchases (e.g., non-randomised virtual items). The data did not support the notion that distress was similarly elevated for all forms of gaming-related purchases. Although participants who spent any money on games, downloadable content for games, or other non-randomised in-game purchases were at slightly higher risk of scoring very high on the Kessler-10, these relative risks were consistently lower than the relative risk for those that purchased loot boxes (see Fig. 1). When these forms of spending were included in a binomial logistic regression with age as a covariate, and gender and dataset as factors (see Table 2), players that purchased loot boxes remained at substantially heightened risk compared to those that did not purchase loot boxes, *RR* = 1.55 [1.26, 1.85], *p* < 0.001. Other forms of video game related spending did not significantly predict distress, though spending on downloadable content was close to significance *RR* = 1.22 [0.99, 1.47], *p* = 0.053 (spending on games, *p* = 0.630, spending on other in-game content, *p* = 0.316). This implies that purchasers of downloadable content may be at slightly higher risk, but further research into this form of spending is required.

**Table 2:** Binomial linear regression results including all forms of measured game spending as factors, with age as a covariate, and gender and dataset as factors.

Predictor	β	Z	Relative risk [95% CI]	*p*
Age^1^	− 0.44	− 7.39	n/a^2^	< 0.001
Gender: female^3^	0.35	3.45	1.29 [1.12, 1.48]	< 0.001
Gender: non-binary^3^	1.09	2.49	2.04 [1.19, 2.92]	0.013
Gender: “prefer-not-to-say”^3^	0.13	0.19	1.10 [0.36, 2.44]	0.850
Gender: other^3^	0.03	0.03	1.02 [0.13, 3.22]	0.979
Dataset	− 0.21	− 1.99	0.86 [0.73, 1.00]	0.047
Games purchased	0.06	0.48	1.04 [0.87, 1.24]	0.630
Virtual items purchased	0.15	1.00	1.12 [0.90, 1.37]	0.316
Downloadable content purchased	0.27	1.93	1.22 [1.00, 1.46]	0.053
Loot box purchased	0.62	4.03	1.55 [1.26, 1.85]	< 0.001

^1^Age converted to Z-score to generate β.

^2^Relative risk inappropriate for age as a continuous covariate.

^3^Comparison group: male.

Research consistently indicates that problem gamblers spend more on loot boxes than non-problem gamblers^11^, and it is well known that problem gambling symptoms are associated with poor psychological wellbeing^29^. We therefore considered next whether the heightened distress observed for players that purchased loot boxes might be due to loot boxes being disproportionately purchased by those with problem gambling symptoms whose heightened risk of severe psychological distress may be due to their problem gambling symptoms and not loot box purchasing. Results of these analyses are shown in Table 3. A binomial logistic regression incorporating demographic characteristics, dataset, and problem gambling classification revealed that problem gamblers were at 2.88 [2.50, 3.25] times the risk of being severely psychologically distressed as non-problem gamblers, *p* < 0.001. However, players that purchased loot boxes remained at substantially heightened risk than non-purchasers when we statistically controlled for problem gambling classification, *RR* = 1.28 [1.05, 1.54], *p* = 0.017. Similar associations for players that purchased loot boxes were observed if raw problem gambling symptom scores, rather than problem gambling classification was employed, with players that purchased loot boxes being at elevated risk relative to those that did not, *RR* = 1.24 [1.01, 1.50], *p* = 0.039. Thus, although problem gambling symptoms explained a substantial amount of the elevated risk of severe psychological distress for players that purchased loot boxes, some elevated risk remained when these symptoms were controlled for.

**Table 3:** Binomial linear regression results including age as a covariate, and problem gambler status, gender, and dataset as factors.

Predictor^1^	β	Z	Relative risk [95% CI]	*p*
Age^1^	− 0.42	− 7.39	n/a^2^	< 0.001
Gender: female^3^	0.49	4.65	1.42 [1.23, 1.63]	< 0.001
Gender: non-binary^3^	1.37	3.10	2.33 [1.44, 3.16]	0.002
Gender: “prefer-not-to-say”^3^	0.22	0.31	1.18 [0.37, 2.57]	0.757
Gender: other^3^	− 0.10	− 0.08	0.93 [0.11, 3.18]	0.933
Dataset	0.01	0.02	1.00 [0.86, 1.16]	0.984
Low-risk gambler^4^	0.09	0.61	1.08 [0.85, 1.35]	0.539
Moderate-risk gambler^4^	0.55	4.11	1.52 [1.26, 1.82]	< 0.001
Problem gambler^4^	1.64	11.10	2.88 [2.50, 3.25]	< 0.001
Loot box purchased	0.34	2.39	1.28 [1.05, 1.54]	0.017

^1^Age converted to Z-score to generate β.

^2^Relative Risk inappropriate for age as a continuous covariate.

^3^Comparison group: male.

^4^Comparison group: Non-problem Gambler.

### Prevalence at various spending rates

**Figure 2:**
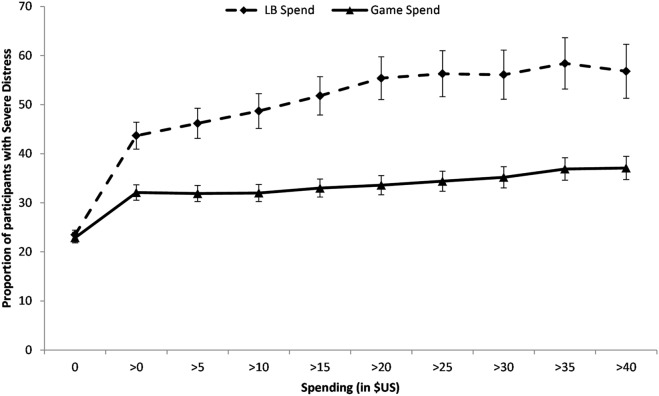
Prevalence of severe psychological distress (K-10 > 30) for those who spent more than a given amount on loot boxes and video games. Error bars represent 95% CIs.

To test whether the association between distress and loot box spending is characterised by a spike in distress associated with a given spending level, or a more gradual positive relationship between the prevalence of severe psychological distress and spending on loot boxes, we plotted Risk Curves for the prevalence of severe psychological distress for participants who reported spending more than a given amount in five dollar increments from no spending until the 75th percentile of spending on loot boxes was reached (beyond the 75th percentile, our participant numbers were too small to provide reliable prevalence estimates). As shown in Fig. 2, the rise in prevalence for severe psychological distress occurs once any spending on loot boxes occurs, and then gradually rises until it asymptotes. There is a much less pronounced increase in risk associated with general spending on video games, again suggesting that the rise in prevalence rates was not due to spending on video games per se. Similar differences were observed between loot boxes and spending on downloadable content and other non-randomised in-game virtual items (see “Supplementary analyses” available online for additional Risk Curves).

## Discussion

Loot boxes have often been likened to traditional forms of gambling. Despite growing evidence that people with problem gambling symptoms tend to spend more on these mechanisms than people without such symptoms, some have argued that further evidence of harm is necessary to justify regulation^19^. Here, we show that individuals that purchased loot boxes were at a moderately heightened risk of experiencing severe psychological distress compared to those that did not purchase loot boxes, based on a standardised psychiatric screening questionnaire. Such heightened distress is typically highly predictive of the presence of bona fide affective disorders in the Australian, Aotearoa New Zealand, and US populations; the populations from which these samples were drawn^20–24^.

Though the results are cross-sectional and correlational, several important analyses help rule out competing explanations for the elevated risk we find for those who purchase loot boxes. First, the heightened risk was present when gender and age were statistically controlled for, suggesting that these demographics were not responsible for the heightened risk. Second, the risks do not appear to be due to any form of game-related spending. Although slightly heightened risk was also present for those who reported spending money on other forms of game-related purchases, this did not explain the differences between those that did and did not purchase loot boxes: differences in relative risk persisted even when the other forms of game-related spending were statistically controlled. Third, the heightened risk associated with loot box purchasing was only partially attenuated when problem gambling status or symptomology were controlled for. Together, these findings suggest that loot box purchasing is associated with unique variance in severe psychological distress.

We cannot rule out reverse causality: those with greater risk of severe psychological distress might be more likely to spend money on loot boxes. However, two important points bear mentioning. First, increased prevalence of severe psychological distress was associated with loot box spending, but not with other game-related spending. This speaks against a “retail therapy” mechanism, where increased distress drives increased hobby-related spending. Such a mechanism would predict general increased spending, rather than a specific increase for spending on randomised items. Second, even if psychological distress was driving loot box purchasing, this would still be concerning. It would imply that a vulnerable group of gamers—those at high risk of bona fide affective disorders—were spending disproportionately on randomised in-game purchases relative to those at lower risk of affective disorders. As analyses were not pre-registered these results should be considered exploratory and replication is therefore required.

An additional consideration is the analysis strategy employed herein—analysing dichotomous outcome variables rather than the continuous measures from which they were computed. Such an analysis strategy undoubtedly results in a decrease in the fidelity of available information. However, we believe the approach is both appropriate and defensible. Although the Kessler-10 provides continuous measures of psychological distress, it also has clearly defined clinical cut-offs that yield high positive predictive value for bona-fide affective disorders^20,23^. Clinical psychology makes clear distinctions between clinical and sub-clinical psychopathology, and although they share similarities, the neurological areas implicated in clinical depression and anxiety appear to be distinct from their sub-clinical counterparts^30^. Thus, we believe the computation of relative risk from established K-10 cut-offs is an appropriate data analysis strategy, especially in the context of examining potential for psychological harms.

Risk Curve analyses identified that the rise in prevalence of severe psychological distress was evident as soon as any loot box spending was reported, with a further gradual rise in prevalence rates as spending increased, until it reached asymptote. This further highlights the potential harm of loot boxes: even small monetary spending on them is associated with increased risk of severe distress. This should give pause to those inclined to argue that the micro-transactional nature of loot boxes, or the typically low spending patterns observed in many studies, render loot boxes harmless. This data pattern may also explain why the relationship between loot box spending and raw psychological distress scores has been relatively modest in previous studies: the rise in severe distress occurred for those spending any money and swiftly reached asymptote, a pattern that would be underestimated by linear analyses^7,8^.

How meaningful are the observed increases in risk likely to be? Our data indicate that, compared to those that did not purchase loot boxes, the relative risk of experiencing severe psychological distress is 1.87 times greater for those that purchased loot boxes. To place this in context, the effects approximate the smallest effect size of interest for media research proposed by Ferguson (though, as Ferguson notes, highly valid dependent measures such as those reported here may imply practical meaningfulness at lower effect sizes^31^). Converting the effect size to Cohen’s *d*^32^ results in *d* of around 0.5, exceeding the half a standard deviation difference some researchers argue is required for participants to feel a difference in well-being^33^. Even when problem gambling symptoms were statistically controlled for, a relative risk of 1.28 [1.05, 1.54] was observed, which suggests that the comorbidity between problem gambling symptoms and loot box spending explains some, but not all of the heightened risk of severe psychological distress for those that purchased loot boxes. It is worth noting that it is also widely acknowledged that smaller effects can accumulate over time^34,35^, although understanding the boundary conditions for this is important^36^.

Researchers have called for a better understanding of the harms associated with loot boxes; providing an important and timely reminder to this field to be appropriately cautious in their claims. It is for this reason that we have repeatedly suggested that an appropriate and cautious approach to regulating loot boxes is the judicious application of consumer warning labels for games containing them^1,7^, or the implementation of spending controls (e.g., limit setting) in games for those at high risk of financial harm (e.g., low income earners)^37,38^. We have previously demonstrated that the value of virtual items to gamers may allow for regulation under existing gambling legislation^16^. However, we remain in favour of a balanced regulatory approach that focuses on providing consumers with the information they need to make informed decisions for themselves and/or their children.

These findings elaborate on a previous meta-analysis showing a reliable relationship between problem gambling symptoms and spending on loot boxes^11^. Further to loot boxes being disproportionately purchased by people with symptoms of problem gambling^11^, they also appear to be disproportionately purchased by people who are psychologically distressed and, by extension, vulnerable. We again call upon industry to take responsibility for protecting vulnerable consumers by implementing appropriate consumer warning labels, and harm minimisation techniques such as spending controls in their games^38^. Further, policy makers should consider the implementation of warning labels on games containing loot boxes to provide consumers with adequate information to make informed decisions about the appropriateness of these mechanisms for themselves and/or their children.

## Supplementary Information

Supplementary Information.

## Data Availability

The datasets analysed during the current study, and copies of the exact analyses are available in the Open Science Framework (OSF) repository https://osf.io/nwkyz/10.17605/OSF.IO/NWKYZ.
