# Supplementary material for: Surprisingly high prevalence rates of severe psychological distress among consumers who purchase loot boxes in video games
Source: Sci Rep. 2022 Sep 27;12:16128. doi: 10.1038/s41598-022-20549-1 (PMC9514709; doi:10.1038/s41598-022-20549-1)
Supplement: Supplementary file 1 — Supplementary Information. [file 41598_2022_20549_MOESM1_ESM.docx]

**Supplementary Analyses**

Below are the additional Risk Curve analyses for spending on Downloadable Content (DLC) and spending on other forms of non-randomised in-game items. The risk curves below are for the prevalence of severe psychological distress for participants who reported spending more than a given amount in five dollar increments from no spending until the 75th percentile of spending on loot boxes was reached (beyond the 75th percentile, our participant numbers were too small to provide reliable prevalence estimates).


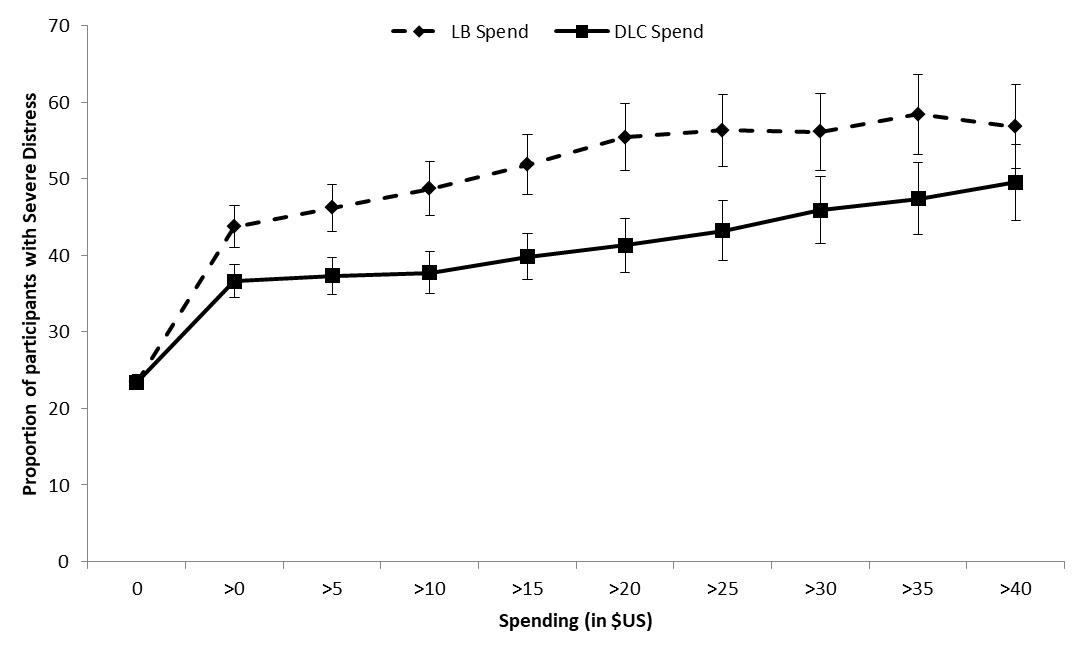


**Figure S1.** Risk Curve analyses for Prevalence of Severe Psychological Distress (K-10 > 30) for those who spent more than a given amount on Loot Boxes and Downloadable Content (DLC). Error bars represent 95% CIs.


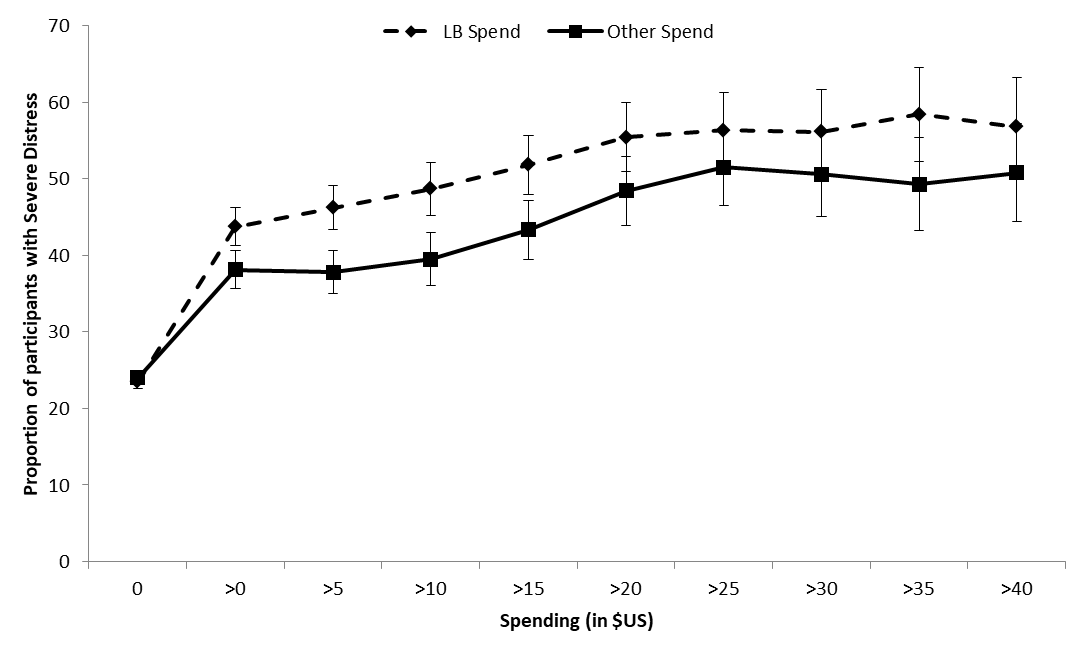


**Figure S2.** Risk Curve analyses for Prevalence of Severe Psychological Distress (K-10 > 30) for those who spent more than a given amount on Loot Boxes and Other non-randomised in-game purchases. Error bars represent 95% CIs.
